# 1,2-Bis(dibromo­meth­yl)benzene

**DOI:** 10.1107/S1600536811050616

**Published:** 2011-12-03

**Authors:** Sin-Kai Fang, Hong-Yi Lin, Kew-Yu Chen

**Affiliations:** a Department of Chemical Engineering, Feng Chia University, 40724 Taichung, Taiwan

## Abstract

In the title compound, C_8_H_6_Br_4_, intra­molecular C—H⋯Br hydrogen bonds generate two *S*(6) rings. The two geminal bromine-atom substituents point to opposite sides of the aromatic ring system. In the crystal, mol­ecules are linked by inter­molecular π–π inter­actions with centroid–centroid distances of 3.727 (9) and 3.858 (9) Å.

## Related literature

For the preparation of the title compound, see: Ghorbani-Vaghei *et al.* (2009 ▶). For its applications, see: Chen *et al.* (2002 ▶, 2006 ▶, 2007 ▶); Chow *et al.* (2005 ▶); Jansen *et al.* (2010 ▶); Pandithavidana *et al.* (2009 ▶); Swartz *et al.* (2005 ▶). For related structures, see: Kuś & Jones (2003) ▶; Qin *et al.* (2005 ▶); Sim *et al.* (2001 ▶). For graph-set theory, see: Bernstein *et al.* (1995 ▶).
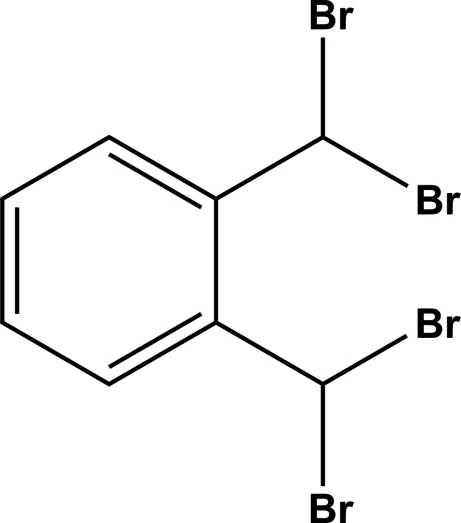


## Experimental

### 

#### Crystal data

C_8_H_6_Br_4_
*M* *_r_* = 421.77
Triclinic, 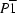

*a* = 7.0222 (8) Å
*b* = 7.7313 (9) Å
*c* = 10.5927 (12) Å
α = 108.473 (10)°
β = 97.108 (9)°
γ = 90.394 (9)°
*V* = 540.61 (11) Å^3^
*Z* = 2
Mo *K*α radiation
μ = 14.83 mm^−1^
*T* = 297 K
0.58 × 0.48 × 0.36 mm

#### Data collection

Bruker SMART CCD area-detector diffractometer
Absorption correction: multi-scan (*SADABS*; Bruker, 2001 ▶) *T*_min_ = 0.122, *T*_max_ = 1.000
4575 measured reflections
2469 independent reflections
1297 reflections with *I* > 2σ(*I*)
*R*_int_ = 0.073

#### Refinement

*R*[*F* ^2^ > 2σ(*F* ^2^)] = 0.091
*wR*(*F* ^2^) = 0.258
*S* = 1.04
2469 reflections
109 parameters
H-atom parameters constrained
Δρ_max_ = 1.62 e Å^−3^
Δρ_min_ = −1.27 e Å^−3^

### 

Data collection: *SMART* (Bruker, 2001 ▶); cell refinement: *SAINT* (Bruker, 2001 ▶); data reduction: *SAINT*; program(s) used to solve structure: *SHELXS97* (Sheldrick, 2008 ▶); program(s) used to refine structure: *SHELXL97* (Sheldrick, 2008 ▶); molecular graphics: *ORTEP-3 for Windows* (Farrugia, 1997 ▶); software used to prepare material for publication: *WinGX* (Farrugia, 1999 ▶).

## Supplementary Material

Crystal structure: contains datablock(s) I, global. DOI: 10.1107/S1600536811050616/aa2031sup1.cif

Structure factors: contains datablock(s) I. DOI: 10.1107/S1600536811050616/aa2031Isup2.hkl

Supplementary material file. DOI: 10.1107/S1600536811050616/aa2031Isup3.cml

Additional supplementary materials:  crystallographic information; 3D view; checkCIF report

## Figures and Tables

**Table 1:** Hydrogen-bond geometry (Å, °)

*D*—H⋯*A*	*D*—H	H⋯*A*	*D*⋯*A*	*D*—H⋯*A*
C8—H8*A*⋯Br1	0.98	2.64	3.364 (16)	131
C8—H8*A*⋯Br2	0.98	2.78	3.420 (16)	124

**Fig. 1.**
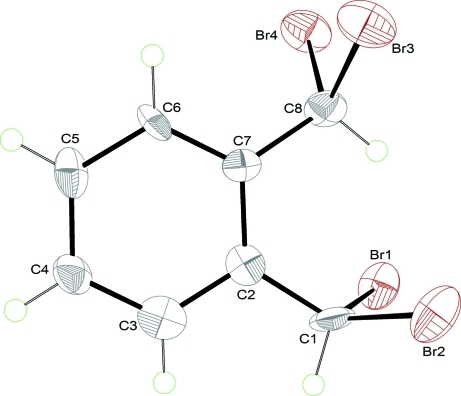
The molecular structure of the title compound, showing 50% probability displacement ellipsoids.

**Fig. 2.**
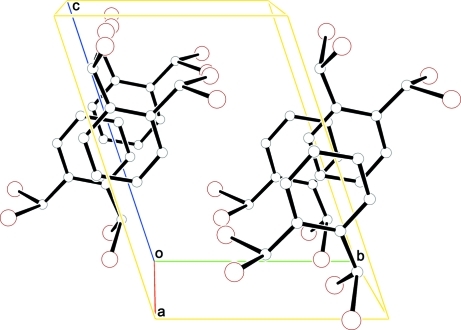
A section of the crystal packing of the title compound, viewed along the a axis. H atoms have been omitted for clarity.

## supplementary crystallographic information

### Comment

The title compound and its derivatives are useful reagents to build a naphthalene ring (Chen *et al.*, 2002, 2006, 2007; Chow *et al.*, 2005; Jansen *et al.*, 2010; Pandithavidana *et al.*, 2009). In addition, they have been prepared as potential precursors to pentacene derivatives (Swartz *et al.*, 2005).

The *ORTEP* diagram of the title compound is shown in Fig. 1. Two intramolecular C—H···Br hydrogen bonds (see Table 1) generate two S(6) ring motifs (Bernstein *et al.*, 1995). The two geminal bromine substituents point to opposite sides of the aromatic ring system.  In the crystal structure (Fig. 2), the molecules are stabilized by intermolecular π–π interactions. Cg1···Cg1^i^ distance is 3.727 (9)Å, symmetry code: (i) -1 - *x*, -*y*, 1 - *z*; Cg1···Cg1^ii^ distance is 3.858 (9)Å, symmetry code: (ii) -*x*, -*y*, 1 - *z*; Cg1 is the centroid of the C2/C7 ring).

### Experimental

The title compound was synthesized by bromination of *o*-xylene with *N*,*N*,*N'*,*N'*- tetrabromobenzene-1,3-disulfonamide in CCl_4_, according to the literature method (Ghorbani-Vaghei *et al.*, 2009). Colorless crystals suitable for the crystallographic studies were isolated over a period of four weeks by slow evaporation from the chloroform solution.

### Refinement

H atoms were positioned geometrically (C—H = 0.93 and 0.98 Å) and allowed to ride on their parent atoms, with *U*_iso_(H) = 1.2*U*_eq_(C)].

### Crystal data

**Table:**

C_8_H_6_Br_4_	*Z* = 2
*M**_r_* = 421.77	*F*(000) = 388
Triclinic, *P*1	*D*_x_ = 2.591 Mg m^−^^3^
Hall symbol: -P 1	Mo *K*α radiation, λ = 0.71073 Å
*a* = 7.0222 (8) Å	Cell parameters from 1856 reflections
*b* = 7.7313 (9) Å	θ = 2.8–29.1°
*c* = 10.5927 (12) Å	µ = 14.83 mm^−^^1^
α = 108.473 (10)°	*T* = 297 K
β = 97.108 (9)°	Parallelepiped, colorless
γ = 90.394 (9)°	0.58 × 0.48 × 0.36 mm
*V* = 540.61 (11) Å^3^	

### Data collection

**Table:**

Bruker SMART CCD area-detector diffractometer	2469 independent reflections
Radiation source: fine-focus sealed tube	1297 reflections with *I* > 2σ(*I*)
graphite	*R*_int_ = 0.073
ω scans	θ_max_ = 29.2°, θ_min_ = 2.8°
Absorption correction: multi-scan (*SADABS*; Bruker, 2001)	*h* = −9→8
*T*_min_ = 0.122, *T*_max_ = 1.000	*k* = −10→10
4575 measured reflections	*l* = −14→14

### Refinement

**Table:**

Refinement on *F*^2^	Primary atom site location: structure-invariant direct methods
Least-squares matrix: full	Secondary atom site location: difference Fourier map
*R*[*F*^2^ > 2σ(*F*^2^)] = 0.091	Hydrogen site location: inferred from neighbouring sites
*wR*(*F*^2^) = 0.258	H-atom parameters constrained
*S* = 1.04	*w* = 1/[σ^2^(*F*_o_^2^) + (0.0875*P*)^2^ + 18.1246*P*] where *P* = (*F*_o_^2^ + 2*F*_c_^2^)/3
2469 reflections	(Δ/σ)_max_ < 0.001
109 parameters	Δρ_max_ = 1.62 e Å^−^^3^
0 restraints	Δρ_min_ = −1.27 e Å^−^^3^

### Special details

**Table:**

Geometry. All s.u.'s (except the s.u. in the dihedral angle between two l.s. planes) are estimated using the full covariance matrix. The cell s.u.'s are taken into account individually in the estimation of s.u.'s in distances, angles and torsion angles; correlations between s.u.'s in cell parameters are only used when they are defined by crystal symmetry. An approximate (isotropic) treatment of cell s.u.'s is used for estimating s.u.'s involving l.s. planes.
Refinement. Refinement of *F*^2^ against ALL reflections. The weighted *R*-factor *wR* and goodness of fit *S* are based on *F*^2^, conventional *R*-factors *R* are based on *F*, with *F* set to zero for negative *F*^2^. The threshold expression of *F*^2^ > σ(*F*^2^) is used only for calculating *R*-factors(gt) *etc*. and is not relevant to the choice of reflections for refinement. *R*-factors based on *F*^2^ are statistically about twice as large as those based on *F*, and *R*- factors based on ALL data will be even larger.

### Fractional atomic coordinates and isotropic or equivalent isotropic displacement parameters (Å^2^)

**Table:**

	*x*	*y*	*z*	*U*_iso_*/*U*_eq_	
Br1	1.0868 (3)	0.1807 (3)	0.90582 (19)	0.0434 (6)	
Br2	0.6361 (3)	0.1787 (3)	0.9231 (2)	0.0492 (6)	
Br3	0.5867 (3)	0.5842 (3)	0.7641 (2)	0.0488 (6)	
Br4	1.0292 (3)	0.5783 (3)	0.7275 (2)	0.0478 (6)	
C1	0.832 (3)	0.078 (3)	0.8116 (17)	0.036 (4)	
H1A	0.8311	−0.0533	0.8003	0.043*	
C2	0.796 (2)	0.094 (2)	0.6741 (16)	0.030 (4)	
C3	0.769 (2)	−0.070 (3)	0.5681 (18)	0.035 (4)	
H3A	0.7804	−0.1803	0.5862	0.042*	
C4	0.725 (2)	−0.073 (2)	0.4342 (17)	0.033 (4)	
H4A	0.7044	−0.1821	0.3639	0.040*	
C5	0.714 (2)	0.097 (3)	0.4101 (16)	0.032 (4)	
H5A	0.6924	0.1004	0.3224	0.039*	
C6	0.735 (2)	0.255 (2)	0.5136 (16)	0.031 (4)	
H6A	0.7183	0.3644	0.4954	0.037*	
C7	0.781 (2)	0.261 (2)	0.6455 (15)	0.024 (3)	
C8	0.821 (2)	0.436 (2)	0.7532 (17)	0.033 (4)	
H8A	0.8533	0.4123	0.8387	0.040*	

### Atomic displacement parameters (Å^2^)

**Table:**

	*U*^11^	*U*^22^	*U*^33^	*U*^12^	*U*^13^	*U*^23^
Br1	0.0330 (10)	0.0605 (15)	0.0388 (10)	0.0108 (9)	−0.0006 (8)	0.0207 (10)
Br2	0.0364 (11)	0.0769 (17)	0.0443 (11)	0.0028 (10)	0.0117 (9)	0.0313 (11)
Br3	0.0386 (11)	0.0406 (13)	0.0671 (14)	0.0169 (9)	0.0072 (9)	0.0165 (11)
Br4	0.0406 (11)	0.0372 (13)	0.0653 (14)	−0.0084 (8)	0.0102 (9)	0.0148 (10)
C1	0.047 (10)	0.027 (10)	0.041 (10)	−0.009 (8)	0.002 (8)	0.024 (8)
C2	0.019 (7)	0.033 (11)	0.033 (9)	0.003 (7)	0.000 (6)	0.008 (8)
C3	0.016 (8)	0.038 (11)	0.051 (11)	0.004 (7)	0.006 (7)	0.014 (9)
C4	0.040 (10)	0.026 (10)	0.033 (9)	0.005 (7)	0.017 (8)	0.003 (8)
C5	0.024 (8)	0.042 (11)	0.025 (8)	0.015 (7)	0.008 (7)	−0.001 (8)
C6	0.042 (10)	0.015 (9)	0.029 (8)	0.006 (7)	0.009 (7)	−0.003 (7)
C7	0.019 (7)	0.026 (9)	0.032 (8)	0.003 (6)	0.007 (6)	0.013 (7)
C8	0.032 (9)	0.030 (11)	0.038 (9)	−0.011 (7)	0.003 (7)	0.014 (8)

### Geometric parameters (Å, °)

**Table:**

Br1—C1	1.965 (17)	C3—H3A	0.9300
Br2—C1	1.932 (19)	C4—C5	1.42 (3)
Br3—C8	2.003 (18)	C4—H4A	0.9300
Br4—C8	1.922 (15)	C5—C6	1.35 (2)
C1—C2	1.49 (2)	C5—H5A	0.9300
C1—H1A	0.9800	C6—C7	1.38 (2)
C2—C7	1.42 (2)	C6—H6A	0.9300
C2—C3	1.40 (2)	C7—C8	1.47 (2)
C3—C4	1.41 (2)	C8—H8A	0.9800
			
C2—C1—Br2	113.8 (12)	C6—C5—C4	120.4 (16)
C2—C1—Br1	112.7 (11)	C6—C5—H5A	119.8
Br2—C1—Br1	110.0 (9)	C4—C5—H5A	119.8
C2—C1—H1A	106.6	C5—C6—C7	122.7 (17)
Br2—C1—H1A	106.6	C5—C6—H6A	118.6
Br1—C1—H1A	106.6	C7—C6—H6A	118.6
C7—C2—C3	119.0 (16)	C6—C7—C2	118.7 (15)
C7—C2—C1	124.9 (15)	C6—C7—C8	120.6 (15)
C3—C2—C1	116.0 (16)	C2—C7—C8	120.7 (14)
C4—C3—C2	121.2 (17)	C7—C8—Br4	112.8 (11)
C4—C3—H3A	119.4	C7—C8—Br3	110.0 (11)
C2—C3—H3A	119.4	Br4—C8—Br3	108.1 (9)
C5—C4—C3	117.9 (16)	C7—C8—H8A	108.6
C5—C4—H4A	121.1	Br4—C8—H8A	108.6
C3—C4—H4A	121.1	Br3—C8—H8A	108.6
			
Br2—C1—C2—C7	61.2 (18)	C5—C6—C7—C8	174.1 (15)
Br1—C1—C2—C7	−65.0 (19)	C3—C2—C7—C6	2(2)
Br2—C1—C2—C3	−117.0 (14)	C1—C2—C7—C6	−176.1 (15)
Br1—C1—C2—C3	116.9 (14)	C3—C2—C7—C8	−175.9 (14)
C7—C2—C3—C4	−1(2)	C1—C2—C7—C8	6(2)
C1—C2—C3—C4	177.2 (14)	C6—C7—C8—Br4	−58.1 (18)
C2—C3—C4—C5	2(2)	C2—C7—C8—Br4	119.7 (14)
C3—C4—C5—C6	−3(2)	C6—C7—C8—Br3	62.6 (16)
C4—C5—C6—C7	5(3)	C2—C7—C8—Br3	−119.6 (13)
C5—C6—C7—C2	−4(2)		

### Hydrogen-bond geometry (Å, °)

**Table:**

*D*—H···*A*	*D*—H	H···*A*	*D*···*A*	*D*—H···*A*
C8—H8A···Br1	0.98	2.64	3.364 (16)	131
C8—H8A···Br2	0.98	2.78	3.420 (16)	124
